# Supplementary material for: An AI-based approach to predict delivery outcome based on measurable factors of pregnant mothers
Source: PLOS Digit Health. 2025 Feb 5;4(2):e0000543. doi: 10.1371/journal.pdig.0000543 (PMC11798466; doi:10.1371/journal.pdig.0000543)
Supplement: Attachment — Submitted filename: Response_to_reviewers.docx [file pdig.0000543.s001.docx]

Dear Sir,

We would like to express our sincere gratitude to you for the opportunity given to submit a revised draft of our manuscript initially titled *‘An AI-based approach to predict delivery outcome based on measurable factors of pregnant mothers’.*

We appreciate efforts spent in providing such an invaluable feedback from your esteemed reviewers. We’re happy to report that reviewer comments from the feedback have largely been addressed and incorporated into the revised manuscript. Where necessary, others have been deleted or modified to meet required criteria.

Response to reviewer comments and concerns are listed below for emphasis.

**Reviewer Comments**

Reviewer #1:

*That sounds a valuable research with interesting findings. Anyhow, i believe the manuscript hasnt been written adhering to the journal`s guidelines; some parts of the manuscript are too lengthy which decreases the readability of the paper. Moreover, it seems there is a need for a precise proof reading of the manuscript in terms of wording and grammar. While the study mentions ethical approval, it does not provide detailed information on how consent was obtained or how ethical considerations were addressed in data handling and analysis. While, the data was collected from a single healthcare facility, which may introduce biases and limit the applicability of the findings to other settings or populations, i reccomend the authors to discuss the socioeconomic and cultural attributes of the population living around the healthcare center in a paragraph or two.*

**Response**: Thank you for the direction. We have made changes to the ethical approval and consent section with additional information that shows which body granted the ethical approval and how patient privacy was protected by emphasizing on the removal of patient identifiers. To help address the second part of the reviewer comment, we have added a sub-section titled sample attributes to highlight sample population characteristics to include population size, cultural and socio-economic make-up of the population.

Reviewer #2:

*The qualitative method mentioned in the manuscript does not seem appropriate, given that the research uses statistical methods. The study primarily relies on machine learning models (Logistic Regression, Random Forest, and Gradient Boosting) to predict delivery outcomes, which are quantitative in nature. These statistical techniques require numerical data and focus on measurable features, like gestational age, maternal blood pressure, and cervical dilation, to make predictions. The research methodology should emphasize quantitative analysis and statistical methods, as those are the primary techniques employed. The mention of qualitative methods appears to be a misalignment.*

**Response**: Thank you for the identification of this misalignment. It is factually accurate that these processes (machine learning or computational and statistical) modeling approaches are quantitative in nature following structured processes for data collection and analysis. All references to **qualitative approach** have been removed.

Additional Clarifications

Extensive review for grammatical errors has been made to improve on the quality of the manuscript. We stand ready to address any further concerns that may come up and look forward to hearing from you in due course with respect to our submission.

Yours Sincerely,

Signed

Michael Owusu-Adjei (corresponding author email: mowusuajdie@st.knust.edu.gh)
