# Supplementary material for: An AI-based approach to predict delivery outcome based on measurable factors of pregnant mothers
Source: PLOS Digit Health. 2025 Feb 5;4(2):e0000543. doi: 10.1371/journal.pdig.0000543 (PMC11798466; doi:10.1371/journal.pdig.0000543)
Supplement: Attachment — Submitted filename: Response_to_reviewers_2.docx [file pdig.0000543.s002.docx]

Dear Sir,

We would like to express our sincere gratitude to you for the opportunity given to submit a revised draft of our manuscript initially titled *‘An AI-based approach to predict delivery outcome based on measurable factors of pregnant mothers’.*

Tireless efforts spent in providing invaluable feedback is appreciated from your esteemed reviewers. Recent reviewer comments from the feedback have been addressed and incorporated into the revised manuscript. Where necessary, descriptions that bring clarity has also been provided.

Response to reviewer comments and concerns are listed below for emphasis.

**Reviewer Comments**

Reviewer comments:

*The reviewers' comments and questions were not appropriately addressed. Table I is a crucial part of the study for understanding the analysis and results. It is titled "Statistical Distribution of Sample Population Counts." The authors appear to have studied women with one or more pregnancies, including those who may or may not have had abortions or stillbirths. However, it is unclear if women with births but no abortions were also included. This lack of clarity makes it difficult to interpret the data.
A single woman could be included in multiple groups; for example, one woman might be in the group of patients with abortions, as well as in the group of abortions with no live births, or abortions with live births. The authors need to revise this table to clarify which groups were compared to obtain the chi-square results they present. As it stands, it is impossible to determine what comparisons were made.
Additionally, ages should be expressed as the mean years ± standard error of the mean.*

**Response**: Thank you for the comment. Request to bring clarity to Table 1 on Statistical Distribution of Sample Population Counts is appreciated. It is indeed true that a single woman could be included in multiple groups in Table 1. However, displayed snapshot used for the analysis in python jupyter notebook indicate that patient records as shown in each row contain no multiple entries. Each row represents a complete record of each patient. The inclusion of Table 1 was to display some statistical information about the sampled population. This snapshot show detailed record of features used. Individual patient records can be found in each row. Each row represents complete records of individual patients.

Detailed description of the sample population used can be found under **Research Materials and Methods.**

Additionally, to bring clarity to Table 1, age range column has been removed. Furthermore, ages have been expressed as the mean years ± standard error of the mean as requested and this can be verified in the **Research Materials and Methods** section. Each of the independent features or variables were compared with the dependent variable (del_type) to determine variable relationship using Chi-sqaure correlation statistic.

We stand ready to address any further concerns that may come up and look forward to hearing from you in due course with respect to our submission.

Yours Sincerely,

Signed

Michael Owusu-Adjei (corresponding author email: mowusuajdie@st.knust.edu.gh)
